# Importance of the Early Recognition of Neonatal Seizure Within Midwifery-Led Birthing Units

**DOI:** 10.7759/cureus.91380

**Published:** 2025-09-01

**Authors:** Saarah Raja, Mithuna Urs

**Affiliations:** 1 Paediatrics and Neonatology, Tunbridge Wells Hospital, Kent, GBR

**Keywords:** arterial ischaemic stroke, midwifery, neonatal, paediatrics, stroke

## Abstract

Perinatal strokes are strokes occurring from 20 weeks of gestation until 28 days of life. Neonatal strokes are part of this spectrum, with the occurrence of stroke from birth up until 28 days of life. Strokes in neonates can lead to future impairments, for example, motor delay, cognitive or speech issues and epilepsy. We report a case of neonatal stroke leading to prolonged seizure activity. We aim to review the importance of early clinical recognition in identifying neonatal strokes in the hope of improving outcomes. We report the case of a baby girl, born at a gestation of 40+6 weeks. The parents first noticed some abnormal movements of the right hand, which self-resolved. There is no further documentation of the nature of these events whilst the baby was at the birthing centre. No subsequent action regarding these concerns was taken at this point. Subsequently, the baby had several further episodes of seizure activity. This included abnormal limb movements, lip smacking, eye twitching and continuous hiccups. MRI confirmed bilateral middle cerebral artery infarctions. On recent examination, no abnormalities were detected, with a normal Moro reflex and good head control. The infant is due for further review and an MRI within the next few months at the time of writing. In conclusion, it is hoped that the case will help highlight the importance of the early detection of neonatal seizures, including an awareness of red flag signs and symptoms.

## Introduction

Perinatal strokes are strokes occurring from 20 weeks of gestation until 28 days of life. Neonatal strokes are part of this spectrum, with the occurrence of stroke from birth up until 28 days of life [1]. These cerebrovascular events are caused by disrupted arterial or venous blood flow within the brain. The areas of disruption may be in one location or multiple locations [2,3]. They can be confirmed by neuroimaging, such as MRI or CT scans. Cranial US may also be used. Neonatal strokes may be divided into two main categories: arterial and venous.

Based on Dunbar and Kirton's classification [3], arterial strokes include arterial ischaemic strokes, where there is 'focal ischemic infarction in one or more arterial territories' [4]. Venous strokes include neonatal haemorrhagic strokes, where there is 'a focal bleed within the brain parenchyma' [4], and cerebral sinovenous thrombosis (CSVT), which 'includes presence of thrombus in one or more cerebral veins or dural sinuses plus parenchymal venous infarction in cerebral venous territory' [4]. In terms of incidence, arterial strokes account for around 80% of strokes. In contrast, around 20% are venous in origin [5]. The global incidence of neonatal strokes is reported as around 20-40 per 100,000 live births [6].

Neonatal strokes are associated with a variety of potential long-term issues. We therefore feel that it is important to raise awareness by reporting on a case of neonatal stroke leading to prolonged seizure activity. We also aim to review the importance of early clinical recognition in the hope of improving outcomes.

## Case presentation

We report the case of a baby girl, born at a gestation of 40+6 weeks. She was born to a 29-year-old primigravida mother at a midwifery-led birthing unit. The baby was born via spontaneous vaginal delivery (cephalic vertex presentation) to non-consanguineous Caucasian parents. The mother had a BMI of 24 at the time of birth, and there were no maternal risk factors. The pregnancy was low risk, and antenatal scans were all normal. Birth weight was 3,540 g. Appearance, pulse, grimace, activity and respiration (APGAR) scores were recorded as 7, 9 and 10 at one, five and 10 minutes, respectively. Condition at birth was noted to be 'good', with no resuscitation required. The baby was screened and treated for neonatal infection due to the possibility of infective causes of seizures, such as meningitis.

During the first day of life, there were concerns regarding feeding. Around 10 hours of life, a newborn and infant physical examination (NIPE) was performed, and no neurological concerns were documented. Around 12 hours of life, whilst still at the birthing centre, the parents noticed some abnormal movements of the right hand, which self-resolved. It was retrospectively documented that the mother verbalised that the baby was having seizures with hand twitching in the morning on the first day of life, but this activity was thought to be due to query hypoglycaemia. It is also documented that the father verbalised that the baby started to have jerking movements in the afternoon on the first day of life. There is no further documentation regarding these events from the healthcare team at the birthing centre. No treatment or management was initiated at this stage.

The baby did not feed until around 12 hours of life. Around 19 hours of life, the baby experienced an episode of duskiness, appearing purple in colour. She desaturated to 53% and was given ventilation breaths, which helped bring saturations up to around 95%. Blood sugar levels were noted to be low at 1.4, so the baby was given 2.5 mL Glucogel (0.5 mL/kg) stat and fed. A decision was made to transfer to the neonatal unit (NNU) at a district general hospital due to low blood sugars and a dusky episode. During transit via ambulance, there was a further episode of duskiness, with saturations of 50% recorded. Ventilation breaths were given again, bringing oxygen saturations up to 99%. Around 20 hours of life, the baby arrived at the hospital. Upon arrival, the baby was noted to have a third episode of duskiness, with apnoea, desaturation to 60% and pallor. Oxygen wafting was given at this point, helping to bring oxygen saturations back up into the 90s. There were also jerking movements of the right hand, followed by the right leg, as well as eye twitching and continuous hiccups, which lasted for about 17 minutes. Immediate management included a loading dose of phenobarbital (20 mg/kg), aciclovir IV, the insertion of two IV lines, IV 10% dextrose (60 mL/kg), benzylpenicillin (double dose) and gentamicin, as well as further blood tests. Cerebral Function Analysing Monitor (CFAM) monitoring was started, and an MRI was requested. The case was discussed within the NNU team at the level 3 unit, who also recommended discussion with the neurology team at a specialist centre the following morning. To summarise, there were two known episodes of seizure activity, with three episodes of duskiness, in the first 24 hours of life.

**Figure 1:**
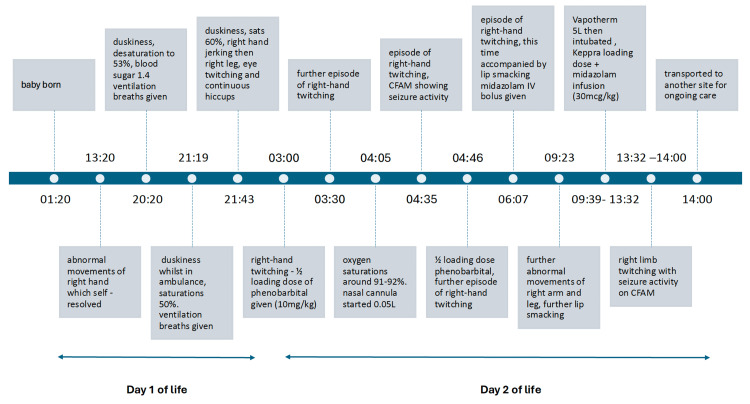
Timeline showing key events from the first 48 hours of life. CFAM: Cerebral Function Analysing Monitor

On day 2, around 26 hours of life, a similar episode was noted with further right-hand twitching. CFAM monitoring showed seizure episodes, and a half loading dose of phenobarbital was given (10 mg/kg). Shortly after, there was a further episode of right-hand twitching. It was noticed that oxygen saturations were around 91%-92%; 0.05 L via nasal cannula was started to maintain oxygen saturations to ≥95%. There was another episode of right-hand twitching with CFAM showing seizure activity, and another half loading dose of phenobarbital was given. Whilst giving this, there was a further episode of right-hand twitching. This episode lasted for around six minutes. Around 29 hours of life, there was another episode of right-hand twitching, this time accompanied by lip smacking. At this point, a midazolam (150 mcg/kg) IV bolus was given. Around 32 hours of life, the neonate is noted to have had further abnormal movements of the right arm and leg, which were 'cycling' and lasted for around seven minutes, as well as further lip smacking. The baby was then commenced on Vapotherm 5 L. After senior review, a decision to give a Keppra loading dose and midazolam infusion (30 mcg/kg) was made. The baby was intubated due to the seizure activity. A size 4.0 endotracheal tube (ETT) was used, and the baby was ventilated at a rate of 50/minute, fraction of inspired oxygen (FiO_2_) 21%, 20/5. Around 36 hours of life, the neonate is noted to have had another episode of right-limb twitching with seizure activity on CFAM. In summary, the baby had a further seven episodes of seizure activity on day 2 of life. A summary of events from the first two days of life can be seen in Figure 1.

The following is a summary of key events whilst ongoing care continued at another hospital. On day 2 of life, midazolam started to be weaned, as the baby remained seizure-free. A decision was made to start regular levetiracetam (Keppra) twice daily. By day 3, midazolam and morphine had been stopped. The cerebral function monitor was also discontinued. On day 4, the baby was extubated onto Vapotherm, which was tolerated well. Keppra (20 mg/kg) was continued twice daily, and the baby remained seizure-free. By day 6 of life, Vapotherm was discontinued, and the neonate was self-ventilating in air. In terms of the septic screen, blood and CSF cultures were found to be negative. Herpes simplex virus (HSV) polymerase chain reaction (PCR) was negative, and aciclovir was discontinued due to this result. Blood cultures were found to be negative, and antibiotics were therefore stopped. Importantly, magnetic resonance venography (MRV), magnetic resonance angiography (MRA) of the head and MRI of the head were performed on day 7 of life.

**Figure 2:**
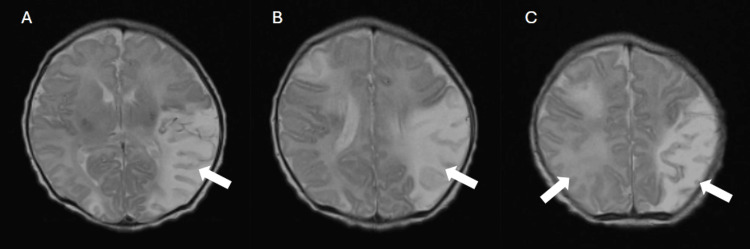
MRI of the head showing areas of infarction within the brain, demonstrated by the white arrows. Images A and B show areas of left middle cerebral artery infarction. Image C demonstrates bilateral middle cerebral artery infarctions, with the left side more affected than the right. These MRI findings place the baby at high risk for a later right-sided motor difficulty. She is also at risk of later mild cognitive and behavioural difficulties and may have an increased risk for later seizures.

MRI findings are as follows, with images displayed in Figure 2: there appears to be a normally formed brain with no evidence of congenital structural abnormality. There is a large area of abnormal signal within the territory of the left middle cerebral artery and a small area within the right middle cerebral artery. There is an associated area of abnormally high signal on T2 in the left thalamus. There is some signal from myelin in the posterior limb of the internal capsule (PLIC), but it is reduced and asymmetrical in appearance. There is an abnormal restriction in the left PLIC. There is a small subdural haemorrhage in the posterior fossa. The aetiology is as follows: these MRI findings are consistent with bilateral middle cerebral artery infarctions. These could be due to an embolic or a vasculitic process. The prognosis is as follows: these MRI findings place the baby at high risk for a later right-sided motor difficulty. She will also be at risk of later mild cognitive and behavioural difficulties and may have an increased risk for later seizures.

The baby returned to the first hospital for ongoing care on day 7 of life. She continued to self-ventilate in air and was given regular doses of Keppra. A nasogastric (NG) tube was in place for feeding. The report findings were explained to the parents, and the plan was for physiotherapy input for as long as needed. Initial physiotherapy assessment noted a preference to look/turn to the right side. Plagiocephaly on the right side and occasional visual fixing not observed to follow through all of the visual field were also noted. Physiotherapists further noted query slightly reduced tone on the right side, with the infant demonstrating active antigravity movements of both upper and lower limbs. Echo was also performed on day 10 of life, with an impression of a normal cardiac study and no cardiac cause for any embolism.

The MRI of the head report findings mentioned above were discussed with a neurological specialist team, who advised an MRA of the neck vessels to be done, as a workup for a possible underlying cause for stroke. This was performed on day 13 of life. MRA findings were as follows: comparison to the previous MRI of the brain shows no significant change in the large areas of established bilateral middle cerebral artery territory infarction, more extensive on the left. There is also a smaller area of infarction in the right occipital lobe, which was not documented on the previous report but is evident in retrospect. The small-volume right infratentorial subdural haemorrhage is unchanged. No new intracranial abnormality is seen.

In the next two days prior to discharge, the baby was roomed in with her parents. She continued to receive regular Keppra twice daily, which was continued on discharge. She was discharged home on day 15 of life.

In terms of follow-up, the specialist neurological team have advised for MRI and MRA ideally before three months of age. The infant has recently been reviewed in the outpatient clinic, at nine weeks of age. On examination, no abnormalities were detected, with a normal Moro reflex and good head control. She is due for further review and an MRI within the next few months.

## Discussion

Seizure activity may be caused by a number of factors such as infection, metabolic disorders and drug withdrawal. It is also important to note the difference between benign jitteriness and seizure activity; jitteriness tends to present as symmetrical movements caused by a stimulus that stop with restraint of the limbs, whereas seizures can be asymmetrical, unstimulated movements that do not cease with restraint. Neonatal strokes are another cause of seizure activity. A study by Martinez-Biarge et al. evaluates potential risk factors for neonatal arterial ischaemic strokes (NAIS) [7]. Infants with NAIS were found to be more likely to have a family history of seizures or neurological disease in comparison to controls. They were also more likely to be male Infants and be born to a primiparous mother. In terms of the presentation of arterial strokes, seizure activity is often present, as shown by this case. Other signs may include periods of apnoea, feeding issues and appearing drowsier than usual [1]. However, some present completely asymptomatically. The gold standard imaging to confirm diagnosis in neonatal arterial ischaemic strokes is MRI [8]. Strokes in neonates can lead to future impairments, for example, motor delay, cognitive or speech issues and epilepsy. There have also been associations with attention deficit hyperactivity disorder and autism [6]. An awareness of these complications highlights the importance of early detection and recognition, as demonstrated by this case.

Gossling et al. have explored key aspects of investigating and managing neonatal seizures [9]. Questionnaires from neonatal staff and paediatric neurologists were used to collect quantitative data, and neonatal seizure guidelines used in different parts of the United Kingdom were analysed. In their 2020 article, they highlight the difficulty in diagnosing neonatal seizures. They further conclude that, although phenobarbital is the traditional first-line treatment, there is wide variation within the United Kingdom in neonatal seizure management. Phenobarbital was identified as the first line due to reasons such as familiarity and local guidelines. Neurologists within the study have suggested levetiracetam as a potential first-line option, and this may be a choice for future management. They believe this medication to be effective at treating neonatal seizures, with minimal side effects [9]. The study identifies some suggestions to improve the detection of neonatal seizures, for example, through greater education on movement patterns suggestive of seizures and further training on neurological assessments of neonates. Another suggestion is the use of video recordings to help improve diagnosis.

## Conclusions

Our report describes a case of prolonged seizure in a neonate, with the first signs of activity not noticed as being of concern. In conclusion, we hope that the case will help highlight the importance of the early detection of neonatal stroke, including an awareness of red flag signs (such as seizure activity and poor feeding). We hope that this will help to prevent missed opportunities based on parental concerns, as demonstrated by this case. The use of telemedicine may be used by remote units or midwifery-led birthing units to obtain specialist input. We also aim to raise awareness of the importance of ongoing training and education within healthcare teams on conditions such as neonatal seizures. In light of this, any early detection of concerning features may be urgently referred to neonatal units or specialists.
